# Immune Response to COVID-19 in India through Vaccination and Natural Infection

**DOI:** 10.26502/fjhs.070

**Published:** 2022-07-29

**Authors:** Tresa Rani Sarraf, Shreyasi Maity, Arjun Ghosh, Suchandan Bhattacharjee, Arijit Pani, Kaushik Saha, Dhrubajyoti Chattopadhyay, Gourisankar Ghosh, Malini Sen

**Affiliations:** 1 CSIR-Indian Institute of Chemical Biology, 4 Raja Subodh Chandra Mullick Road, Kolkata 700032; 2 Biobharati Life Sciences, EN-35, Sector V, Kolkata 700091; 3 University of California, San Diego, 9500 Gilman Drive, La Jolla, CA 92093; 4 Sister Nivedita University, DG 1/2 New Town, Kolkata 700156

**Keywords:** Covishield, Covaxin, COVID-19, RBD-WT and RBD-DELTA, SARS-CoV-2

## Abstract

In India, COVID-19 (Corona Virus Disease-2019) continues to this day, although with subdued intensity, following two major waves of viral infection. Despite ongoing vaccination drives to curb the spread of COVID-19, the relative potential of the administered vaccines to render immune protection to the general population and their advantage over natural infection remain undocumented. In this study, we examined the humoral and cell-mediated immune responses induced by the two vaccines Covishield and Covaxin, in individuals living in and around Kolkata, India. We also compared the immune responses induced separately by vaccination and natural infection. Our results indicate that although Covishield generates a better humoral immune response toward SARS-CoV-2, both vaccines are almost equivalent in terms of cell-mediated immune response to the virus. Both Covishield and Covaxin, however, are more effective toward the wild-type virus than the Delta variant. Additionally, the overall immune response resulting from natural infection in and around Kolkata is not only similar to that generated by vaccination but the cell-mediated immune response to SARS-CoV-2 also lasts for at least ten months in some individuals after the viral infection.

## 1. Introduction

COVID-19 (Corona Virus Disease-2019) is caused by infection with the Severe Acute Respiratory Syndrome causing Corona Virus – 2 (SARS-CoV-2), a single strand RNA virus [1; 2]. The disease is believed to have originated in China in December 2019 [3; 4]. Since that time, COVID-19 has ravaged several countries causing many fatalities. The World Health Organization (WHO) designated COVID-19 as a pandemic in March 2020 [1]. So far, there have been multiple major SARS-CoV-2-associated infection waves worldwide. India suffered from two such waves, the first one spanning July – November 2020, and the second one spanning March – June 2021.

SARS-CoV-2 enters the human host usually through the upper respiratory tract and binds to the ACE2 receptor expressed on host epithelial cells through its Spike protein - Receptor Binding Domain, the S-RBD [2; 5]. The S-RBD – ACE2 interaction disrupts the normal function of ACE2, which is essential for the maintenance of proper human physiology [6]. The ensuing reactions to the establishment of the viral infection in the human host range from standard inflammation-causing mild disease to uncontrolled cytokine storm, which may even lead to death [7; 8]. Several studies have indicated that blockade of the activity of the S-RBD domain of the virus would be effective in restricting disease progression. These studies have paved the way toward the development of several vaccines against the Spike protein of the virus, an example being the Oxford–AstraZeneca chimpanzee adenovirus vectored vaccine ChAdOx1 nCoV-19 (AZD1222) [9]. The same vaccine is known as Covishield in India. Conventional vaccines against the whole virus have also been developed to fight off infection, Covaxin (product of Bharat-Biotech, India) being an example [10].

The SARS-CoV-2 infection has resulted in many hospitalizations and deaths in India. With the dipping of the first COVID wave, a second wave, which emerged with few mutant forms of the SARS-CoV-2 virus, led to even more hospitalizations and deaths than the first wave. The severity of the second wave in India is evident from the two population-based studies we conducted. In a rural area with ~4000 people, no hospitalization or death occurred during the first wave of infection (between August and November 2020), although nearly 50% of the same population tested antibody positive in January-February, 2021. The same population had four hospitalizations and one death during the second wave (March-June, 2021). Additionally, in a low-bed-capacity hospital in Kolkata, the numbers of hospitalizations/deaths in three months during the first wave (September to November 2020) were 30 (hospitalization) and 0 (death). In contrast, the numbers were 54 (hospitalization) and 9 (death) over a period of only 31 days during the month of May in 2021. These numbers highlight the severity of COVID-19 during the second wave.

A major mutant (DELTA variant) with L452R and T478K double mutations in the S-RBD domain dominated the second infection wave [11; 12]. With the fag end of this wave still continuing, the danger of a third wave looms. Although a major vaccination drive has begun, only about 55% of the massive Indian population has so far been fully vaccinated (two vaccine doses). While several studies targeted numerous vaccines to determine their efficacy against several SARS-CoV-2 strains [13], no comparative studies have been done on the two vaccines most used in India, especially with respect to natural infections mediated by the wild type virus (Wuhan-HU-1 isolate) and the DELTA variant. In this scenario, it is very important to evaluate and compare the efficacy of the currently administered vaccines, Covishield and Covaxin, particularly with reference to the immune protection generated by natural infections. To this end, we undertook a comprehensive study aimed at assessing the immune response profiles prevalent in the general Indian population of West Bengal, using blood samples from vaccinated, naturally infected, and unvaccinated (apparently healthy) cohorts. Our goal was to evaluate the levels of the immune response against the wild-type virus and the DELTA variant in the different study groups, and thereby analyze and compare the immune protection generated through vaccination and natural infection.

In a prior population-based epidemiological study centered on West Bengal, we had demonstrated about 90% effectiveness of the Covishield vaccine in generating antibody response (humoral immune response) against wild-type SARS-CoV-2 [14]. Here, we extended our study to compare the effectiveness of the Covishield and Covaxin vaccines in terms of both humoral and cell-mediated immune responses to the wild-type (WT) and mutant (DELTA) variant(s) of SARS-CoV-2. Moreover, we evaluated the potency of both vaccines in relation to the potential immune protection rendered by natural infection.

## 2. Methods

### 2.1 Human Subjects & Ethical Declaration

An Institutional Review Board (IRB) on Human Subjects was constituted following the guidelines of the Indian Council for Medical Research (ICMR). The IRB read and discussed the research proposal and provided a certificate of approval dated December 20, 2020, to Biobharati Life Sciences for the research conducted in this study. Samples were collected from donors following the protocol approved by the IRB after receiving their signed consent. Gram Panchayat (Village Council) leaders along with the local non-government organizations helped assemble donors in the rural places and were present during sample collection. In Kolkata, donor groups were assembled by various non-government organizations. The human subjects belonged to five different donor groups: vaccinated with Covishield (147 participants), vaccinated with Covaxin (42 participants), naturally infected from the first COVID wave (16 participants), naturally infected from the second COVID wave (14 participants), and uninfected apparently healthy individuals (19 participants).

### 2.2 Isolation of blood plasma and PBMC

Plasma and PBMC isolation was performed following published protocols, with minor modifications [15; 16]. Approximately 10 ml of blood collected in a vial (BD Vacutainer Cat. No. 368856) from each donor was centrifuged at 200xg to separate the plasma. The collected plasma was again centrifuged at 1000xg to remove platelets and subsequently stored at −80°C until used for antibody measurement. The remaining blood was diluted in a 1:1 ratio with PBS and carefully layered on Histopaque for density gradient centrifugation at 350xg for 20 minutes without brakes. Subsequently, the buffy coat (comprising PBMC) was collected in a separate tube and washed 2 times with PBS by centrifugation at 350xg for 8 minutes each time to remove residual histopaque. To remove traces of platelets the last wash was done at 200xg for 10 minutes. The final PBMC pellet was resuspended in 1 ml PBS for hemocytometer counting. Following centrifugation again at 350xg for 8 minutes, isolated PBMC were frozen by resuspension in cold FBS with 10% DMSO. 6 × 10^6^ cells/ml were aliquoted in each cryovial and immediately stored in Mr. Frosty freezing container at −80°C. After 24 hours cryovials were transferred from −80°C to liquid nitrogen until used for cell-mediated immune response experiments.

All blood samples were transferred on ice with a maximum time gap of 2.5 hours from the time of collection. The samples were processed for cryopreservation of PBMC in our laboratory immediately after arrival. Sera harvested from a few drops of blood were collected in fresh tubes, heated for 15 min at 60 °C, and saved at −80 °C until further use for antibody measurement.

### 2.3 Measurement of total Spike-RBD protein-specific antibodies (IgG/IgM/IgA)

ELISA was performed following the method published by Stadlbauer D et.al [17] with some modifications using a test kit developed in-house. Briefly, each well of 96 well high binding ELISA plates (Corning, USA) was coated with 100 ng of antigen (S-RBD wild type or Delta variant) followed by 16 hours incubation at 4°C and subsequent washing with TBST. All antigen-coated wells were blocked with 5% non-fat dry milk (Himedia, India) dissolved in TBST for 1 hour at room temperature and again washed with TBST before the addition of sera (plasma) at 1:100 dilution. Following incubation for 90 minutes at room temperature and subsequent washing with TBST, a secondary antibody (goat, anti-human IgG/IgM/IgA-HRP conjugated; Invitrogen, Cat#A18847) in 1:5000 dilution was added to each well and incubation at room temperature was continued for 45 minutes. Following washing with TBST, the plate reactions were developed according to the standard TMB method [17], and OD was measured at 450 nm on the iMark microplate reader (BioRad). Pure C-terminally poly-histidine tagged RBD-WT (Cat # 40592-V08H) and RBD-DELTA (Cat#40592-V08H90) used in the assay were expressed in HEK293 cells and obtained from Sino Biological US Inc.

We also performed a titration of the well-studied SARS-CoV-2-specific human monoclonal antibody (mAb B38) [18] against S-RBD using the identical ELISA method to validate the assay using plasma. B38 monoclonal antibody was purchased from Invivogen (Cat# cov2rbdc2-mab1).

### 2.4 Competition assay to monitor RBD: ACE2 inhibition

We performed ELISA to estimate the amount of S-RBD-specific antibody in blood plasma or sera that is capable of blocking the interaction between RBD and ACE2 and reported that as percent inhibition, based on published protocol [23]. Pure C-terminally poly-histidine tagged RBD-WT and RBD-DELTA were used to coat high binding ELISA plates (Corning, USA). 100 ng of antigen (S-RBD; same for WT and DELTA variant) was coated on each of 96 wells of the ELISA plates. Coated plates were incubated for 16 hours at 4°C followed by washing with TBST and subsequently blocked with 5% non-fat dry milk (Himedia, India) dissolved in TBST before incubation for 1 hour at room temperature. A 100 uL mix of 1:10 diluted plasma (or sera) with 20 ng of human angiotensin-converting enzyme-2 (ACE-2) (Cat # 10108-H05H, Sino Biological US Inc.) was then added to each well, and incubation was continued for 90 minutes at room temperature before washing again 4 times with TBST. Since human ACE-2 expressed in HEK293 cells is tagged with mouse Fc at the C-terminus, an anti-mouse IgG-HRP secondary antibody (Sigma USA), was added to each well at 1:5000 dilution, after washing. Following 45 minutes of incubation at room temperature and again washing with TBST, the plates were developed according to the standard TMB method, and OD was measured at 450 nm in BioRad iMark microplate reader five minutes after the reaction was stopped (reading A). Since mouse secondary antibody also cross-reacts with human antibody to a certain extent, a parallel ELISA was carried out identically with only ACE2 omitted (reading B). In another reaction, 20 ng ACE2 in 100 uL TBST was incubated with each RBD coated well followed by washing, secondary antibody incubation, and development (reading C). OD_450_ in C represents the maximum binding of 20 ng ACE2 to coated RBD. OD_450_ in A represents ACE2 remaining after plasma/sera competition. OD_450_ in B represents the cross-reactivity of a human antibody to a mouse secondary antibody. Percent inhibition was calculated using the formula [100-(A-B)/C X 100] [23]. All reactions were done at least in duplicates, and several were triplicated. We found that some seronegative samples also showed inhibition. However, this inhibition never exceeded 15%. Therefore, effective competitors are those, which showed greater than 15% inhibition. To validate the authenticity of the competition assay, we performed a competition assay between ACE2 and mAb B38 instead of sera for RBD-WT binding.

### 2.5 Ni-NTA affinity pulldown assay to test RBD-ACE2 or RBD-antibody binding in the context of antibody competition with ACE2 for RBD

To visualize specific binding competition between plasma antibody and ACE2, we used the Ni2+ pull-down assay. In this case, 400 ng poly-histidine fused RBD-WT or RBD-DELTA was bound to Ni2+ NTA beads (10 uL) and incubated with positive or negative sera (500 uL of 1:10 dilution) as determined by ELISA, or ACE-2-Fc (400 ng) in a buffer containing 150 mM NaCl, 1% Tween20, 25 mM Tris-HCl 7.5. Binding was allowed to occur for 1 hour, followed by extensive washing with the same buffer (500 uL) 4 times. After washing the bead was treated with 20 uL of 2X SDS loading buffer, followed by separation of the bound protein by SDS-PAGE and staining with Coomassie Blue. The competition binding was done identically except both ACE-2 and plasma were mixed with ACE-2 bound beads. Control reactions were also done where RBD was eliminated in the binding reaction. To determine the identity of the antibody band captured by His-RBD, we performed a pulldown assay with pure mAb B38 as a control

### 2.6 Estimation of cell mediated immune response

Cell mediated immune response was evaluated following published protocols [15; 16; 19]. For all, assays cells were thawed in prewarmed water (37°C) and diluted in 10 ml pre-warmed RPMI media (10% FBS, 1% Penicillin, Streptomycin, 1% l-glutamine). Following brief centrifugation, cells were cultured in 10 ml RPMI media for 18 hours under normal tissue culture conditions and counted using a hemocytometer. Subsequently, cells were plated in 24 well plates at 2.4× 10^5^ cells /well and incubated with 2 µg/ml of either the wild type or the mutant version (L452R-SRBD) of SARS-CoV-2 S-RBD for 24 hours, with Brefeldin added into the culture medium for the last 4 hours. For each protein batch, just the protein diluent (PBS with 30% glycerol) was used as the vehicle control. Protein incubation was followed by one wash with PBS, fixation with 1% paraformaldehyde for 10 minutes at room temperature, 3 washes in PBS, and storage at 4°C overnight. The next day PBMC were permeabilized with 0.1% Tween 20/PBS for 10 minutes at room temperature, washed in permeabilization buffer, and stained with Anti-human CD4 (PE, Biolegend), Anti-human CD8 (APC, Biolegend), Anti-human IFNγ (PE-CY7, Biolegend) and Anti-human CD154/CD40L (Alexa fluor 700, Biolegend), for 1 hour at 4°C. After washing once each with permeabilization buffer and PBS, PBMC were resuspended in PBS for acquisition on BD LSRFortessa Cell Analyzer. Analysis was performed using FCS Express 5 software.

### 2.7 Data analyses

The cutoff was determined using sera from 11 control subjects known to be uninfected from our previous study. The mean of the OD values of the negative control replicate plates plus three times the standard deviation of the OD value distribution gave the cutoff. P-values of post-COVID samples were calculated by the Kruskal-Wallis test followed by Dunn’s multiple comparison analysis. Graph Pad-Prism software was used for the calculations.

## 3. Results

### 3.1 Experimental strategy:

We strategized an investigative plan for substantiating a prior study focused on the SARS-CoV-2 infection rate in West Bengal, India, and extending it further for comparing the extent of immune response to SARS-CoV-2 infection and vaccination. Earlier, we had reported that the infection rate during the first infection wave with the original SARS-CoV-2 (WT) virus, in the population in and around Kolkata, India was roughly 40% [14]. Other reports also found that a large fraction of the Indian population (~40%) was infected during the first wave of infection [20; 21]. Subsequently, the second wave of infection, mediated mostly by the DELTA variant, hit the city’s population starting around the end of March 2021 and lasted till the end of June. By the end of July 2021, a substantial fraction of the city’s population received two doses of vaccines approved in India, - Covishield (ChAdOx1; also known as Oxford-Astrazeneca) and Covaxin (BBV152; originated in India). In this study, we focused on measuring the humoral immune response (level of antibody inhibiting ACE2-RBD interaction) and T cell-mediated immune response of the naturally infected (both first wave and second wave) and vaccinated population.

To this end, we organized five experimental groups (Table 1). The first two groups comprised the population vaccinated with either Covishield (1) or Covaxin (2). These two groups had no known history of infection, but some of them could have been naturally infected without any symptoms. Groups 3 and 4 were naturally infected either during the first wave (3) or during the second wave (4). Group 3 had developed antibodies against S-RBD (WT) by the end of December 2020 but were mostly asymptomatic [14]. They were not tested by RT-PCR. Their blood samples were collected again in July 2021 for the current study. Individuals in Group 4 were infected from April-June 2021. All Group 4 individuals were symptomatic, and most were hospitalized. They tested positive for RT-PCR. Blood samples were collected from them post-recovery. The last group (Group 5) served as a reference or control. The subjects in Group 5 had no known history of infection and were not vaccinated. Some in this group were periodically tested by RT-PCR and they always tested negative. However, some of them could have been infected asymptomatically.

In order to estimate humoral and cell-mediated immune response to SARS-CoV-2 generated through vaccination and natural infection, blood samples were collected from all designated groups as explained in Table 1. In all the samples, the titer of anti-S-RBD antibody (IgG/IgM/IgA) against both the WT and DELTA variant-specific S-RBD proteins and its potential to block ACE2-S-RBD interaction was evaluated by virtue of antigen-antibody interaction. The antigens S-RBD WT and S-RBD DELTA are hereafter referred to as RBD-WT and RBD-DELTA, respectively. Furthermore, we analyzed the cell-mediated immune response toward RBD-WT and RBD-DELTA within a subgroup (Supplementary Table 1) of each group of samples because not many individuals were willing to donate the required volume of blood needed for the required assays. Cell-mediated immune response was confirmed by quantifying the IFNγ and CD40L levels of CD8 and CD4 lymphocytes.

### 3.2 Estimation of humoral immune response to SARS-CoV-2 elicited through vaccination and natural infection

*Levels of antibody detecting RBD-WT and RBD-DELTA among the test groups:* We determined the antibody titer against the virus in all the plasma samples under study by ELISA. We then determined the cut-off OD values for both RBD-WT and RBD-DELTA using sera that were previously shown to be negative for SARS-CoV-2 [14]. The cut-off was found to be 0.25 for both WT and DELTA (Supplementary Figure 1A-C).

**Figure 1:**
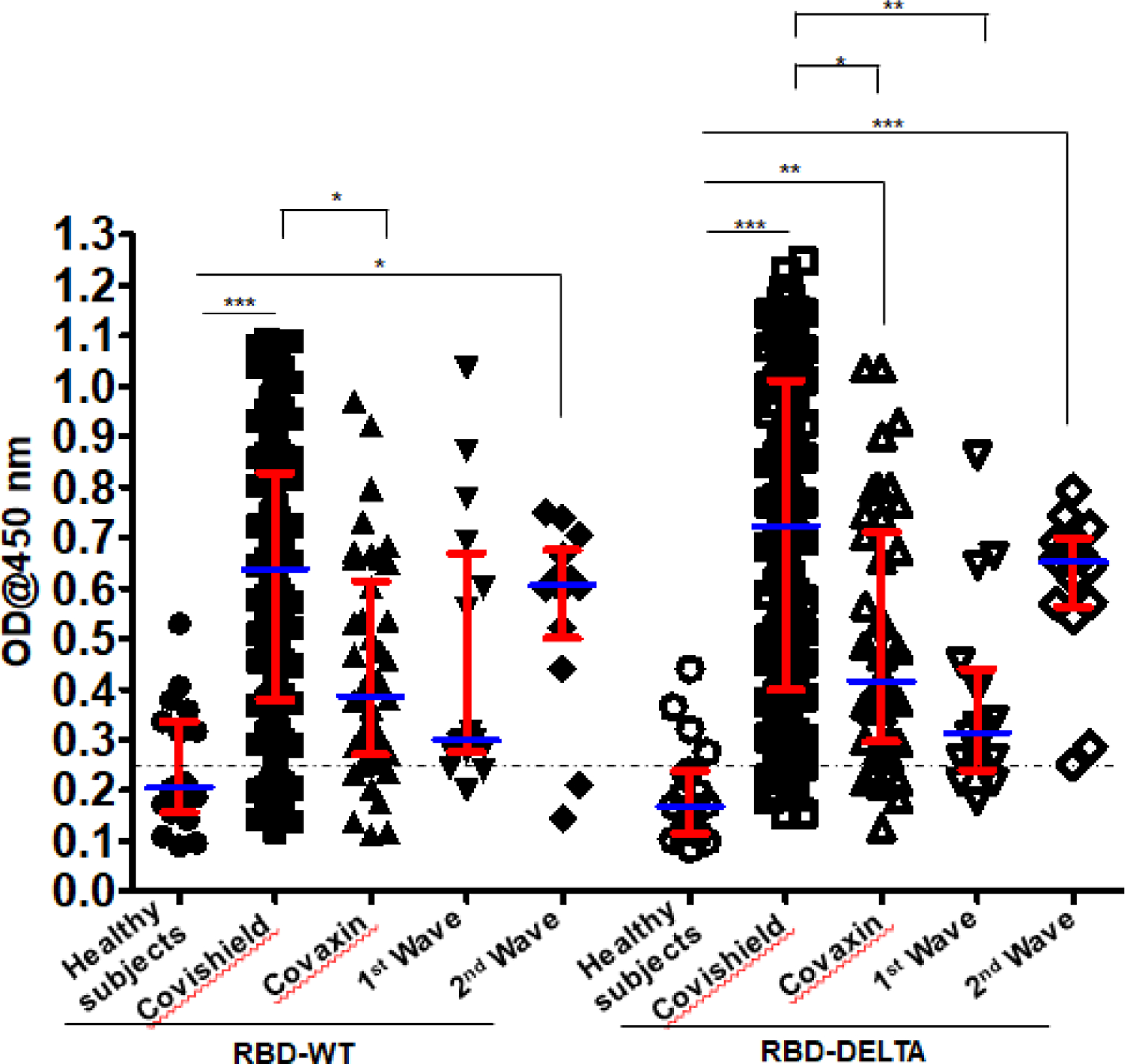
Seroreactivity of the infected and vaccinated population against Spike (S)-RBD. Seroreactivity against RBD-WT and RBD-DELTA of all samples in each of the five groups was analyzed. Kruskal-Wallis test (**P<0.0001**) followed by Dunn’s multiple comparison analysis was performed to compare the seropositivity of vaccinated (Covishield and Covaxin) and naturally infected (first wave and second wave) individuals against RBD-WT and RBD-DELTA, using healthy subjects as reference. Scatter plots show lines at the median with error bars representing the interquartile range. P values equal to or less than 0.05 were considered statistically significant. Blue lines represent the median and red bars represent the interquartile range.

We examined the validity of the ELISA method to measure seropositivity by using a monoclonal antibody termed mAb B38. Clone B38 was isolated from a COVID-19 patient and it was subsequently shown to neutralize SARS-CoV-2, *in vitro,* by blocking the interaction between the SARS-CoV-2 S-RBD and the host receptor ACE2 [18]. As expected, we found that mAb B38 binds to the S-RBD-coated plate higher than 1000X antibody dilution (Supplementary Figure 1D). Figure 1 shows the sero-sensitivity for all samples tested against both RBD-WT and RBD-DELTA. Corroborating our previous finding [14], Covishield showed about 86% efficacy in generating anti-RBD-WT antibodies, i.e. about 86% of the volunteers tested turned out seropositive after vaccination.

Using similar assay conditions, Covaxin showed slightly less efficacy than Covishield (81%). Both the first and second infection waves also generated anti-wild type S-RBD antibody in the infected population, with about 81% (first wave) and about 86% (second wave) of the volunteers showing seropositivity. The antibody profile of the same cohorts against the RBD-DELTA protein was similar except that 100% of second wave samples were seropositive.

This is not surprising since all of them were tested by RT-PCR and were infected within a narrow window of time during the peak of the second wave with mostly DELTA infection (Table 1). It should be noted that all first wave samples were seropositive against RBD-WT from December 2020 to January 2021. As shown in Supplementary Figure 1E, nearly all samples showed waning S-specific antibody with a couple even below the cut-off. This is not surprising since these subjects were infected anytime between July and October of 2020, about 8–11 months before the time of the second collection.

### 3.3 Levels of antibody blocking RBD-WT or RBD-DELTA binding with ACE2 among the test groups:

**Figure 2:**
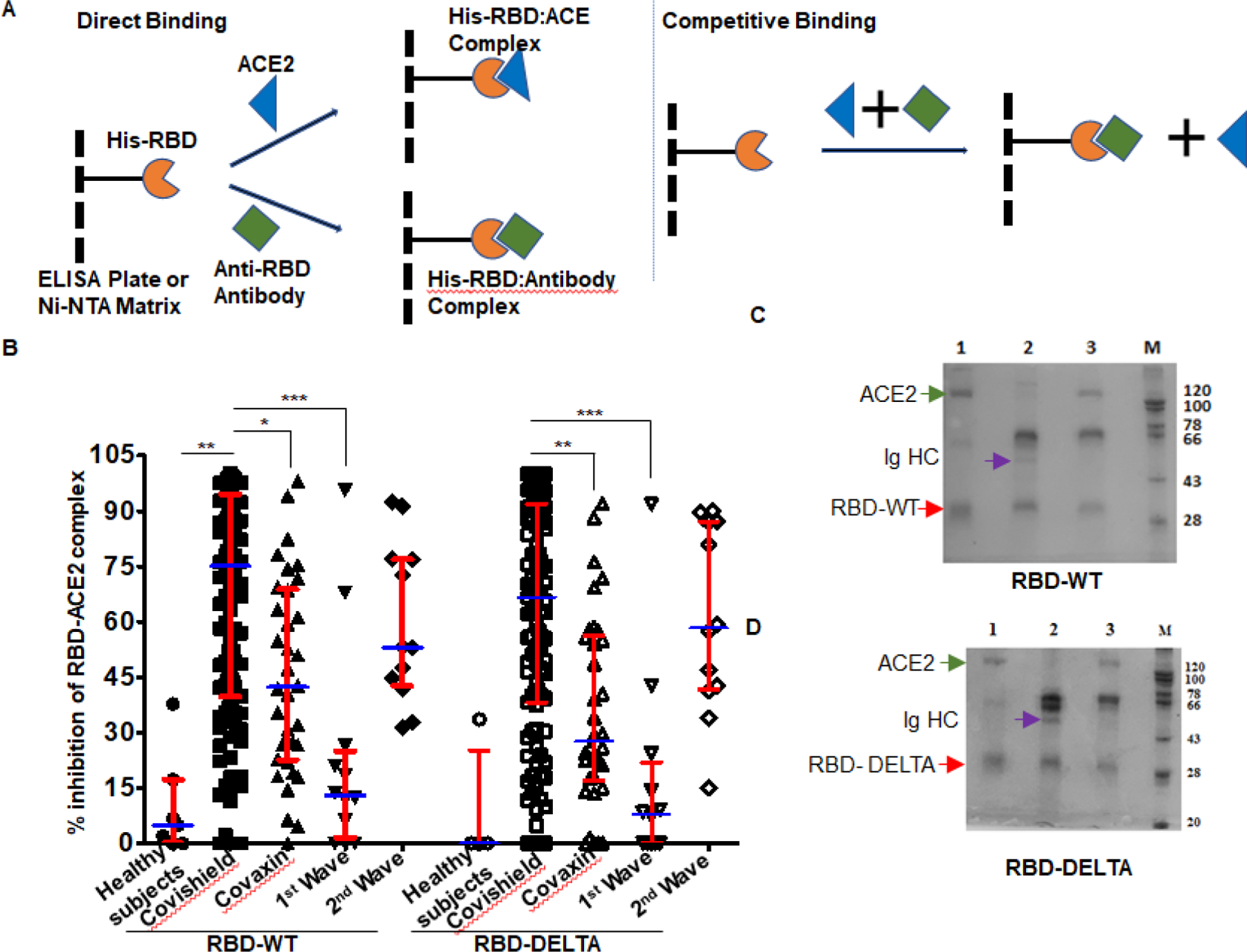
Competition between plasma/sera from infected and vaccinated people and ACE2 for RBD binding as surrogate neutralization activity. A. A schematic representation of direct binding between His-RBD-WT (or His-RBD-DELTA) bound to Ni-NTA beads (or ELISA plate) and RBD-specific antibody or ACE2 (left). In the competition assay, both plasma/sera and ACE2 are added to the Ni-NTA beads (or plate), and plasma/sera containing RBD-specific antibodies outcompete ACE2 for RBD-WT or RBD-DELTA binding (right). B. Percent signal inhibition of ACE2-RBD binding by sera/plasma samples (1:10 dilution) from five different groups. 100 ng His-RBD-WT (left) or His-RBD-DELTA (right) was used as the capture antigen for sera/plasma and 20 ng ACE2 and inhibition of ACE2 - RBD binding by sera/plasma was estimated by ELISA. Each sample was tested in duplicate with each dot representing a mean of two readings. Competition assays of each sample for both RBD-WT and RBD-DELTA were done in parallel on the same plate for better reproducibility. Kruskal-Wallis test (**P<0.0001**) followed by Dunn’s multiple comparison analysis was performed to compare the blocking potential of the plasma samples from the vaccinated and infected groups, using healthy subjects as reference. Scatter plots show lines at the median with error bars representing the interquartile range. P values equal to or less than 0.05 were considered statistically significant. C. Competition was tested using the Ni-NTA affinity pulldown assay. His-RBD-WT (400 ng) was captured by Ni-NTA beads (red arrow) bound pure ACE2 (400 ng: green arrow) (lane 1). A sample with an RBD-specific antibody efficiently competed for ACE2 binding (lane2) but a sample negative for an RBD-specific antibody did not (lane 3). ‘M’ denotes MW standards. The violet arrow denotes Ig heavy chain that is specifically bound to RBD-WT. D. Same as ‘C’ except RBD-DELTA was used as capture antigen. Positive and negative plasma used here were the same as those used in C.

In order to test the ability of anti-RBD antibodies to block ACE2-RBD interactions, we adapted assays from published reports [22; 23]. These reports confirmed that in vitro inhibition of interactions between RBD and ACE2 by RBD-specific antibodies correlates strongly with the neutralization of live or pseudo-virus infection of human cell lines. The schematic of our assay is shown in Figure 2A. We first quantified the efficiency of interaction between ACE-2 and RBD-WT or RBD-DELTA by ELISA. Indeed, ACE2 bound to both RBDs with nearly equal efficiencies, with 100% binding saturation at ~50 ng ACE2 (Supplementary Figure 2A). The blocking potential of the ACE2: RBD interaction by seropositive plasma/sera samples was evaluated by adding a mixture of ACE2 (20 ng) and test plasma/sera to either WT- or DELTA-RBD coated wells (explained in the method section). To validate the competition assay, we tested if pure mAb B38 can effectively compete with ACE2 for binding RBD-WT. Indeed, titration experiments showed mAb B38 inhibiting the binding of 20 ng ACE2 in a concentration-dependent manner (Supplementary Figure 2B). We found that not all seropositive samples were effective in inhibiting the ACE2: RBD interaction *in vitro*. For RBD-WT, whereas high blocking efficiency was observed in Covishield, Covaxin, and second-wave seropositive samples (84%, 79%, and 85% respectively), in the first wave samples blocking efficiency was relatively low (61%) (Figure 2B). For the DELTA variant, both vaccinated cohorts produced relatively lower levels of blocking antibodies – Covishield (81%) and Covaxin (71%). The second wave cohort showed similar protection against DELTA as WT RBD (85%). However, the first wave cohort showed even further diminished activity for DELTA (47% versus 61%) for reasons explained above (Figure 2B and Table 1). We noted that a significant fraction of S-specific antibodies that detect DELTA lacked the potency to inhibit ACE2-DELTA interaction. The blocking efficiency is mostly correlated with the antibody titer; the greater the antibody titer, the greater the ability to block the ACE2: RBD interaction. In fact, most of the plasma samples from the first wave group with antibody titers barely above the cut-off were not effective in blocking the RBD: ACE2 interaction unlike those that had high titers. Following the same trend, the mean antibody titer and ACE2-RBD interaction blocking potency of plasma samples from Covaxin vaccinated individuals were lower than those from Covishield vaccinated individuals. Quite interestingly, a few of the plasma samples collected from the apparently uninfected and unvaccinated donors also showed some degree of seropositivity and ACE2 blocking potential, although at a much lower level, implying the presence of cross-reactive antibodies against S-RBD overlapping antigenic epitopes [24; 25]. It is also possible that these individuals could have harbored asymptomatic infections.

To further validate the ELISA assay and corroborate the presence of RBD-WT- and RBD-DELTA-specific blocking antibodies, we demonstrated both interactions between RBD and ACE2, and competition between ACE2 and specific antibodies for RBD binding, using a second inhibition assay. Herein, we performed the affinity pull-down assay followed by an SDS-PAGE analysis. Since both RBD-WT and RBD-DELTA used for this assay are poly-histidine tagged, the proteins were initially bound to Ni-NTA beads, which were then subjected to treatment with either anti-S-RBD positive or negative plasma or pure ACE2. Accordingly, in the competition assay, when ACE2 was added to the Ni-NTA beads bound to RBD-WT or RBD-DELTA in the presence of plasma containing S protein-specific antibodies we found a drastic reduction in ACE2 binding, with concomitant binding of Ig heavy chain to the beads. As expected, plasma with no S protein-specific antibody failed to compete for ACE2 binding (Figure 2C & D, compare lanes 2 and 3). To positively confirm the antibody bands in sera, we used B38 mAb in the pulldown assay. As shown in Supplementary Figure 2C, S-RBD retained two bands migrating similarly for both B38 mAb and the seropositive samples. These two bands correspond to the Ig heavy chain (HC) and light chain (LC). This assay was used to test five more positive samples from three groups: Covishield, the first wave, and the second wave. They all showed antibody-dependent inhibition of the RBD: ACE2 complex formation (Supplementary Figure 2D). ACE-2 protein was retained by His-RBD in the absence of positive sera (Supplementary Figure 2D, comparing lanes 3 versus lanes 4 to 8). Supplementary Figure 2E shows that ACE2 and RBD-specific Ig binding to the bead is require the presence of RBD.

Overall, these results suggest that while natural infection with SARS-CoV-2 is as effective as vaccination in generating an antibody response, this humoral immune response may not last long enough to fight off future infections. Whether the currently administered vaccinations will provide long-term humoral immunity against the virus is a matter that only time will confirm.

### 3.4 Evaluation of cell-mediated immune response to SARS-CoV-2 generated through vaccination and natural infection.

T cell activation and cytokine expression being an important facet of immunity [16; 19; 26; 27; 28], we focused on the level of T cell response to vaccination and natural infection, in the general population of West Bengal. Accordingly, blood samples collected from the same groups of volunteers in the specified time frame as explained in the previous section were tested for CD8+ and CD4+ T cell immunity against both the RBD-WT and RBD-DELTA proteins. Since IFNγ is an established cytokine for estimating both CD8+ and CD4+ T cell activation in response to viral antigens [19; 26], we evaluated the levels of intracellular IFNγ in the CD8+ and CD4+ T cells of a subgroup of individuals in each of the 5 groups summarized earlier upon stimulation with either the RBD-WT or the RBD-DELTA protein (Table 1 and Supplementary Table1). Alongside, we also examined the level of expression of CD40 ligand (CD40L/CD154) on account of its documented role in the activation of antigen-specific CD4+ T cells through interactions with the CD40 receptor expressed on antigen-presenting cells [34]. The idea was to estimate the recall immune responses generated in cells expected to be primed through vaccination or natural viral infection, after stimulation with viral antigen *ex vivo* [16; 29; 30; 31; 32]. We used S-RBD protein as the antigenic stimulant, thus accounting for antigen processing as well as its presentation in the estimation of recall immune response [19; 32].

**Figure 3:**
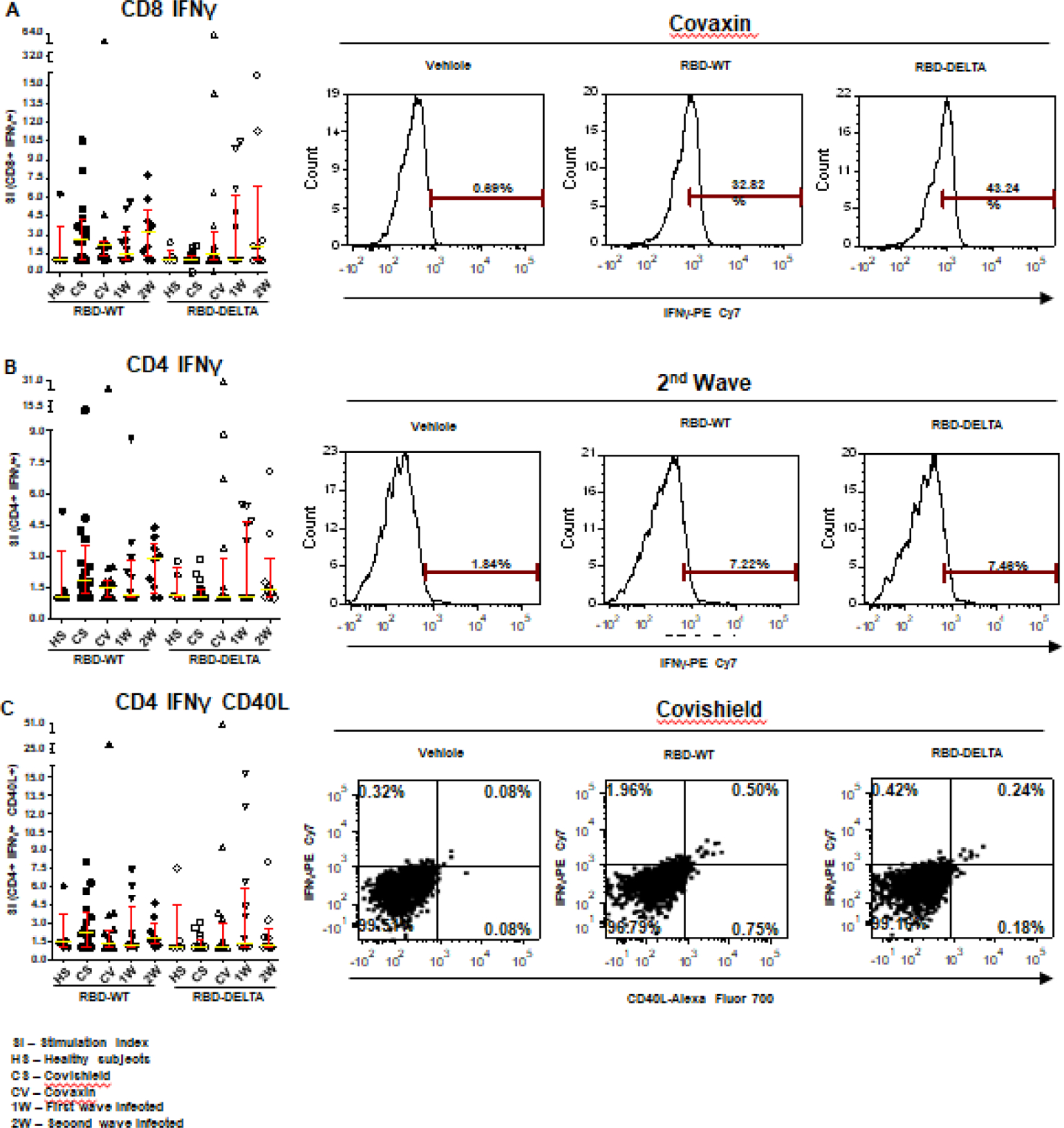
S-RBD protein-specific T cell response in different groups of individuals. S-RBD-specific T cell response was compared between vaccinated (Covishield or Covaxin), naturally infected, and healthy individuals by estimation of Stimulation index (SI) by flow cytometry. Panel A: Graph represents CD8^+^ IFNγ^+^ T cell response in vaccinated (Covishield or Covaxin), naturally infected, and healthy individuals after stimulation with RBD-WT and RBD-DELTA protein. The stimulation index (SI) was calculated by dividing the antigen (S-RBD) specific CD8^+^ IFNY^+^ response with the corresponding vehicle control (50% glycerol) response. Kruskal-Wallis test (**P=0.0310**) followed by Dunn’s multiple comparison analysis was performed to compare the SI of the individuals from different groups. Scatter plots show lines at the median and error bars represent the interquartile range. A representative histogram shows the IFNγ^+^ CD8 T cell frequency in the stimulated sample compared to vehicle control in a vaccinated (Covaxin) individual. Highly positive IFNγ^+^ cells were considered for analysis. Marker gates are based on vehicle control. Panel B: Graph represents CD4^+^ IFNγ^+^ T cell response in different groups of individuals. SI was calculated as described above after Kruskal-Wallis test (**P=0.3436**) with Dunn’s multiple comparison analysis. FACS representation shows IFNγ^+^ CD4 T cells in a naturally infected individual during the second wave. Panel C: Graph represents SI of IFNγ^+^CD40L^+^ CD4^+^ T cells. Kruskal-Wallis test (**P=0.3816**) followed by Dunn’s multiple comparison analysis was performed to compare the SI of the individuals from different groups. FACS quadrant plots represent the double-positive (IFNγ^+^CD40L^+^) CD4 T cells in a vaccinated (Covishield) individual after stimulation with RBD-WT and RBD-DELTA protein.

Increases in CD8 and CD4 T cell-specific IFNγ and CD40L in response to the S-RBD protein were analyzed by fluorescence activated cell sorting (FACS) of the PBMC harvested from blood samples with the use of appropriate fluorophore-tagged antibodies, as explained in Materials and Methods. The gating strategy for this analysis with the incorporation of Fluorescence Minus One control is illustrated in Supplementary Figure 3. Analysis of the recall response to antigen stimulation in terms of IFNγ+ or CD40L+ CD8 and CD4 T cells was based on rigorous FACS gating of antigen-specific high IFNγ/CD40L producers as explained in the same figure. In this study, as summarized in Supplementary Table 1, we included 16 individuals each from the two vaccinated groups (Groups 1 & 2), 12 and 9 individuals, respectively, from the naturally infected groups (Groups 3 & 4), and 5 individuals, who had undetectable levels of anti-S-RBD neutralizing antibody from the control group (Group 5). Antibody levels and neutralization (blocking antibody) efficacy of these subjects are shown in Supplementary Figure 4A-D. In the FACS analysis of each PBMC sample of each group, the change in the level of T cell activation after stimulation with the S-RBD protein was assessed with respect to the corresponding ‘no protein’ control, i.e. vehicle control (VC) and depicted as Stimulation Index (SI), with the FACS representation of specific examples from both the vaccinated and natural infection groups (Figure 3).

The RBD-specific CD8+IFNγ+, CD4+IFNγ+, and CD4+IFNγ+CD40L+ responses (SI values) in the vaccinated and naturally infected groups as compared to the healthy subjects are depicted in Panels A, B, and C of Figure 3 respectively. Although the differences in responses obtained across the different groups did not turn out statistically significant following multiple comparison analyses after the Kruskal-Wallis test, considerable response, especially that of CD8+IFNγ+ was detectable in several individuals. While Covishield and Covaxin yielded similar CD8+IFNγ+ response to RBD-WT (>60% tested individuals with SI above 2), CD8+IFNγ+ response yielded by the two vaccines to RBD-DELTA was lower. Covaxin, however, fared better than Covishield (31% vs. 6% of individuals with SI above 2). The CD8+IFNγ+ response of the naturally infected groups, on the other hand, was roughly the same to both RBD-WT and RBD-DELTA, with the CD8+IFNγ+ response of the second wave group being almost in line with that of the two vaccines to the wild type protein (Panel A). The CD4+IFNγ+ and CD4+IFNγ+CD40L+ responses, although detectable in some individuals across the different groups, were much less than the CD8+IFNγ+ responses (Panels B and C), perhaps on account of inconsistencies in the number of healthy cells after cryopreservation. Only the second wave natural infection group and the Covishield group showed more than 50% response (50% individuals with SI above 2) in terms of CD4-IFNγ and CD4-IFNγ+CD40L respectively. The relatively low T cell response seen in the control group could be because of the presence of cross-reactive T cells, which is not an uncommon phenomenon [33]. Taken together, with the limited number of samples tested, it appears that Covaxin and natural infection could potentially generate a better T cell immune response than Covishield against infections with the mutant virus.

## 4. Discussion

Escalating fatalities from worldwide dissemination of SARS-CoV-2 infection and designation of COVID-19 as a pandemic led to extensive research on understanding the virus and avenues to restrict viral infection and the spread of disease. Several exemplary research articles have ever since documented the mode of viral transmission, the rise of different mutant variants, and the various safety measures needed for protection from infection. With the marvelous effort put forward by a few companies, several vaccines have also been developed. The currently ongoing vaccination drives worldwide are now aimed at controlling COVID-19. Yet, after two major waves of infection and deaths, COVID-19 persists. Moreover, much remains unknown about the level of protective immune response generated through natural infections by SARS-CoV-2, and the efficacy of vaccinations in different communities all over the globe.

We previously published the rate of SARS-CoV-2 infection and the efficacy of the Covishield vaccine in generating anti-S-RBD antibody in several pockets all over West Bengal, India [14]. In the current article, we have evaluated the potential of the antibodies generated by both Covishield and Covaxin to block viral infection using an in vitro assay [23], focusing on individuals in and around Kolkata, India. We have also analyzed the efficacy of Covishield and Covaxin in generating T cell-mediated immune response, a major arm of immune memory that is crucial for combating viral infections [16; 17; 18; 19; 20]. Additionally, we have compared the immune response generated by vaccinations with that developed through natural infections. As a marker of cell-mediated immune response generated through vaccination and natural infection, we have used T cell-associated IFNγ and CD40L in line with the involvement of these molecules in sustaining the recall immune response generated upon antigenic stimulation of already primed T cells [16; 19; 26; 29; 30; 34; 35].

In this study we demonstrated a large fraction of the blood plasma samples collected from vaccinated individuals to be effective in blocking the interaction between RBD-WT and ACE2, Covishield-specific plasma being considerably more effective than Covaxin-specific plasma in this respect (Figures 1 & 2). Both groups were also effective against the DELTA virus. Although there was no p-value, DELTA neutralization was slightly less efficient than neutralization of WT. The ability of the S-specific antibodies of both vaccines to block the ACE2-RBD-DELTA interaction was the same as that projected in other reports [36; 37; 38].

These reports showed that 84% and 80% of subjects with two-dose Covisheild and Covaxin vaccine, respectively, could neutralize DELTA as opposed to 81% and 71%, respectively, in our study. Natural infection (second wave) was as effective as Covishield in generating blocking antibody titers. The paucity of blocking antibodies among individuals belonging to the first-wave infection group may be due to the dearth of long-lived antibody-secreting plasma cells [39]. In terms of T cell-mediated immune response (Figure 3 & Supplementary Figure 3) the picture is quite different. T cell-mediated immune responses generated by the two vaccines toward the wild-type protein were quite similar, although Covaxin could be potentially better than Covishield for the DELTA variant.

Natural infection, on the other hand, appeared to be quite similar to both the vaccines with regard to the wild-type virus, but potentially better than Covishield with regard to the DELTA variant. Moreover, T cell-mediated immunity from the first infection wave lasted for as long as ten months, indicating the persistence of memory T cells in one-time infected individuals [16; 17]. The observed lack of correlation between antibody-driven immune response and cell-mediated immune response in the vaccinated groups could be due to the difference in the frequency of available antigenic epitopes for B cells and T cells in the two groups.

A few recent studies showed vaccines were significantly less protective against DELTA variant than the WT virus [40; 41]. These studies used non-pathogenic helper virus systems for the neutralization test, whereas we used a purely protein-protein interaction-based assay. Both assay methods are artificial, not mimicking the true infection in a real-life scenario. The best answer to the effectiveness of a vaccine against DELTA and other variants, perhaps, will come from a real-time dependent population-based study. Several studies have already suggested that WHO-approved vaccines are quite effective against all the variants currently present in the air since a significantly lower number of individuals have been severely sick or hospitalized after two doses of the vaccines [42; 43; 44; 45]. This trend is also similar in India [12]. Hospitalizations and deaths after vaccination here have so far been associated with severe co-morbidities.

This study comes with certain limitations in the perspective of similar studies, on account of some technical drawbacks. The inconsistency in sampling across the different groups that was apparent in the unevenness in gender distribution, age, and COVID severity was due to the unavailability of the required numbers of appropriate volunteers, despite the joint effort of several non-government organizations. Timing and funding issues, moreover, precluded the detection of CD3 positive T cells in FACS and the use of peptide pools instead of S-RBD protein, for antigen recall response, thus dampening the readout of our assay somewhat.

However, despite limitations, our study reveals that among the human subjects under consideration, both Covishield and Covaxin generate humoral and cell-mediated immune responses against WT and DELTA SARS-CoV-2. Although the vaccines vary in the levels of the immune responses they generate, both are effective. Since most countries are now requiring only fully vaccinated people to travel and only WHO-approved vaccines are recognized, our study provides assurance to the international vaccine authority of the positive effect of the vaccines administered in West Bengal, India. However, whether Covishield/Covaxin vaccinations or natural infection will generate enough long-term immunity to cross the hurdle of future infections by the SARS-CoV-2 wild-type strain or its mutant variants remains a question, more so, on account of instances of inappropriate memory T cell activation [46].

## Supplementary Material

1

## Figures and Tables

**Table 1:** Summary of test subjects for five different groups included in the study. IQR= Interquartile range.

Group	Total	Male	Female	Age, Median (IQR)	Days after 2 doses vaccination / infection, Median (IQR) or Mean**	Days between 1st and 2nd dose of vaccination, Mean***	Seropositive WT/DELTA (%)	*Blocking positive WT/DELTA (%)
Covishield	147	94	53	58 (49.75–65.00)	103 (54–113)	84	86.39/93.88	83.67/80.95
Covaxin	42	17	25	52 (42.00–60.00)	72 (38–107)	28	80.95/85.71	78.57/71.43
1st Wave	16	4	12	30 (25.00–41.00)	275**	NA	81.25/75.00	60.00/46.67
2nd Wave	14	7	7	47 (32.00–67.00)	71**	NA	85.71/100.00	85.71/85.71
Healthy subjects	19	13	6	27 (23.00–44.00)	NA	NA	36.84/21.05	26.32/5.26

* Only the seropositive subjects were tested for blocking ACE2:RBD interaction. The time of infection of the first wave subjects is somewhat speculative since none of them were tested by RT-qPCR. They were all infected before December 2020, and an assumption was made based on the peak of the first wave of infection in India and projected as mean (**). The peak of the second wave of infection, projected as mean (**) was a narrow range between April and June 2021. The duration between 1^st^ and 2^nd^ dose of vaccination is projected as mean (***). All second wave subjects were tested by RT-qPCR. Subjects infected during the second wave were more severely affected than those infected during the first wave. NA: Not applicable
