# Supplementary material for: Immune Response to COVID-19 in India through Vaccination and Natural Infection
Source: Fortune J Health Sci. Author manuscript; Available in PMC 2023 Jun 10. (PMC10257406; doi:10.26502/fjhs.070)
Supplement: 1 [file NIHMS1855913-supplement-1.pdf]

related symptoms, hospital admissions, and mortality in older adults in England: test negative case-control study. BMJ 373 (2021) n1088.

46. Thieme CJ, Anft M, Paniskaki K, Blazquez-Navarro A, Doevelaar A, Seibert FS, et al. Robust T Cell Response Toward Spike, Membrane, and Nucleocapsid SARS-CoV-2 Proteins Is Not Associated with Recovery in Critical COVID-19 Patients. Cell Rep Med 1 (2020) 100092.

This article is an open access article distributed under the terms and conditions of the Creative Commons Attribution (CC-BY) license 4.0

| RBD-WT | | | | |
|---|---|---|---|---|
| | Mean | SD | 3XSD | Mean + 3XSD = Cut Off |
| 0.187 | 0.146636 | 0.036319 | 0.108956 | 0.256 |
| 0.171 | | | | |
| 0.109 | | | | |
| 0.095 | | | | |
| 0.141 | | | | |
| 0.09 | | | | |
| 0.155 | | | | |
| 0.172 | | | | |
| 0.14 | | | | |
| 0.2 | | | | |
| 0.153 | | | | |

A

| RBD-DELTA | | | | |
|---|---|---|---|---|
| | Mean | SD | 3XSD | Mean + 3XSD = Cut Off |
| 0.114 | 0.136318 | 0.039494 | 0.118482 | 0.255 |
| 0.124 | | | | |
| 0.102 | | | | |
| 0.098 | | | | |
| 0.230 | | | | |
| 0.189 | | | | |
| 0.128 | | | | |
| 0.113 | | | | |
| 0.133 | | | | |
| 0.129 | | | | |
| 0.145 | | | | |

B

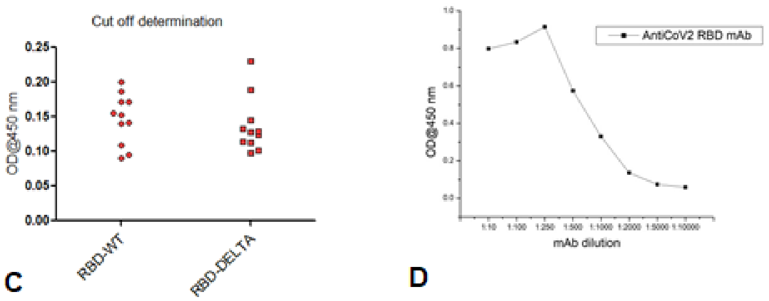


C D

| Sl No | $A_{450}$ in December | $A_{450}$ in June |
|---|---|---|
| 1 | 0.343 | 0.317 |
| 2 | 1.1035 | 0.778 |
| 3 | 0.5355 | 0.692 |
| 4 | 0.8285 | 0.6 |
| 5 | 1.069 | 1.035 |
| 6 | 0.467 | 0.284 |
| 7 | 1.213 | 0.561 |
| 8 | 1.35 | 0.283 |
| 9 | 1.095 | 0.299 |
| 10 | 0.923 | 0.3 |
| 11 | 0.774 | 0.87 |
| 12 | 0.887 | 0.273 |
| 13 | 0.646 | 0.238 |
| 14 | 0.409 | 0.2 |
| 15 | 0.5 | 0.242 |

E

**Supplementary Figure 1:** Cut off value determination using 11 negative sera samples collected previously. A. $A_{450}$ reading of sera against the RBD of the spike protein of WT virus (RBD-WT) using ELISA. The cut off value was calculated from the mean and standard deviation. B. $A_{450}$ reading of sera against the S-RBD-DELTA and the cut off value using ELISA. C. $A_{450}$ distribution plot using the program graphpad. D. Binding of serially diluted B38 mAb to

S-RBD by ELISA. E. Comparison of antibody titer against S-RBD the 15 first wave samples taken six months apart (December 2021) and (June 2021).

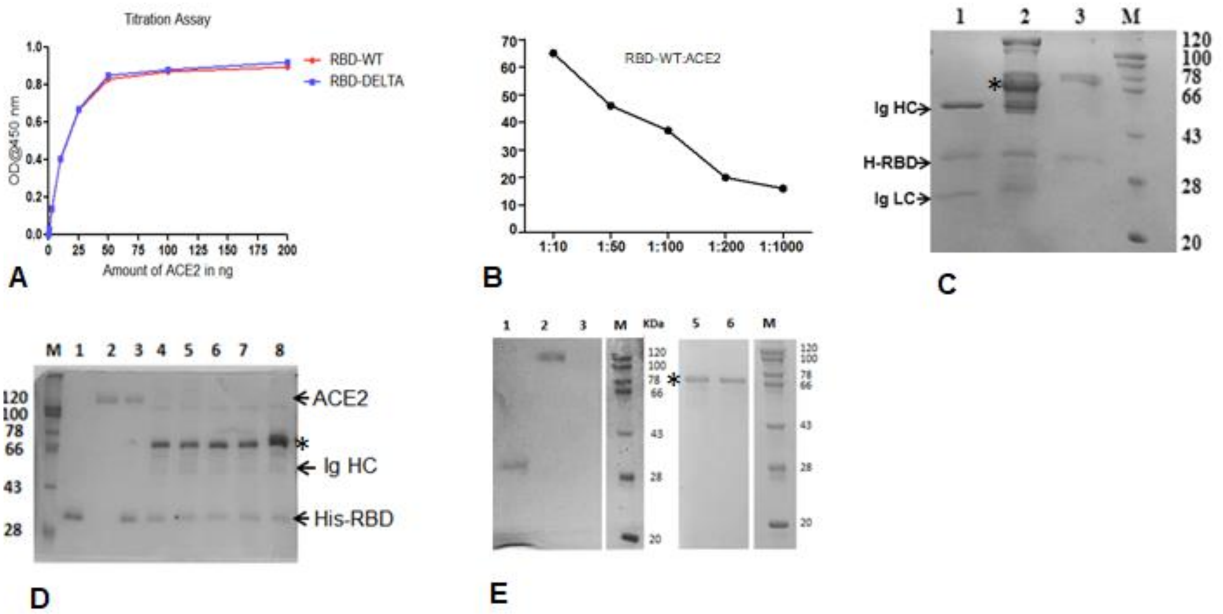


**Supplementary Figure 2:** Interactions between S-RBD and ACE2 or Specific Ig. **A**. ELISA-based binding titration where increasing concentrations of ACE2 protein was added to wells coated with 100 ng RBD-WT (red) or RBD-DELTA (blue). RBD proteins were prepared as poly-histidine fusion proteins and ACE2 was prepared as a Fc fusion protein. **B.** Percent inhibition of the RBD:ACE2 complex by the mAb B38 using the program Graphpadprism. **C**. Binding interaction between His-RBD-WT and pure mAb (B38) or Spike (S) protein-specific antibody in plasma (seropositive and neutralization positive) or seronegative plasma by His-pulldown assay followed by SDS-PAGE separation and staining with Coomassie blue. His-RBD-WT pulled down both heavy and light chains of IgG (Ig HC and Ig LC) of mAB (B38) (lane 1), seropositive/neutralization antibody (IgG (lane 2) but no IgG heavy chain could be seen in seronegative sample (lane 3). **D**. Competition between RBD-WT and ACE for S-RBD specific antibody binding using the Ni2-NTA affinity pulldown assay. Lanes, M) MW standard, 1) His-RBD-WT, 2) ACE-2, 3) ACE2 captured by His-RBD-WT, 4) +ve sera (Covishield), 5) +ve sera ($2^{nd}$ Wave), 6) +ve sera ($2^{nd}$ Wave), 7) +ve sera ($1^{st}$ wave), 8) +ve sera ($1^{st}$ wave). **E.** Recombinant ACE2-mFc and antibody in sera/plasma do not interact with Ni2+-beads. Lanes, 1) RBD-WT incubated with Ni-NTA, 2) Only ACE2 (loading control), 3) ACE2 incubated with Ni-NTA, M) MW standard, 5) Crude +ve sera incubated with Ni-NTA, 6) Crude –ve sera incubated with Ni-NTA. The prominent band, denoted by an *, seen in all pulldown samples incubated with sera/plasma non-specifically binds to beads.

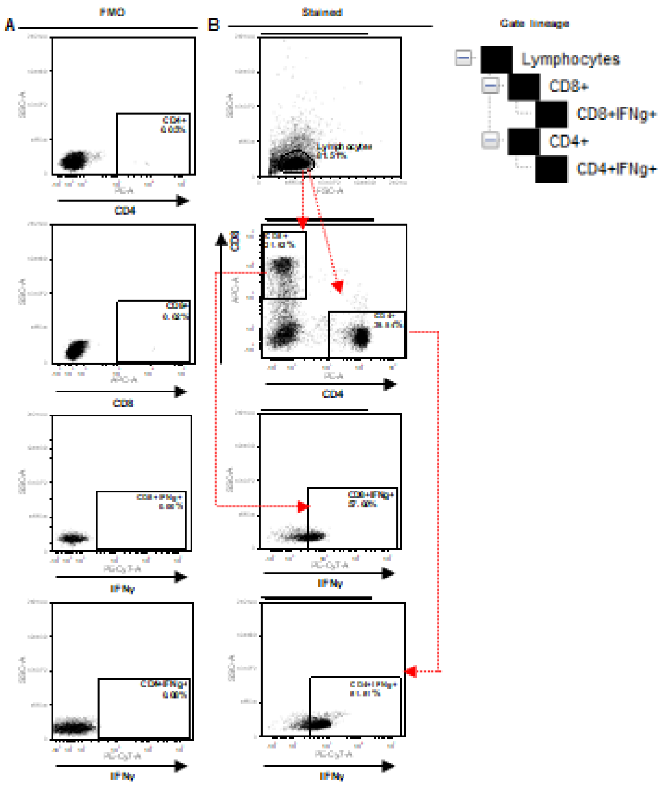


**Supplementary Figure 3.** Depiction of FACS gating of PBMC collected from human blood samples. A. Representation of Fluorescence Minus One (FMO) control for CD8, CD4 and IFNg. B. Initial gating on lymphocytes followed by demarcation of CD8+IFNg+ and CD4+IFNg+ positive populations, as explained in gate lineage.

 *DOI: 10.26502/fjhs.070~~55~~*

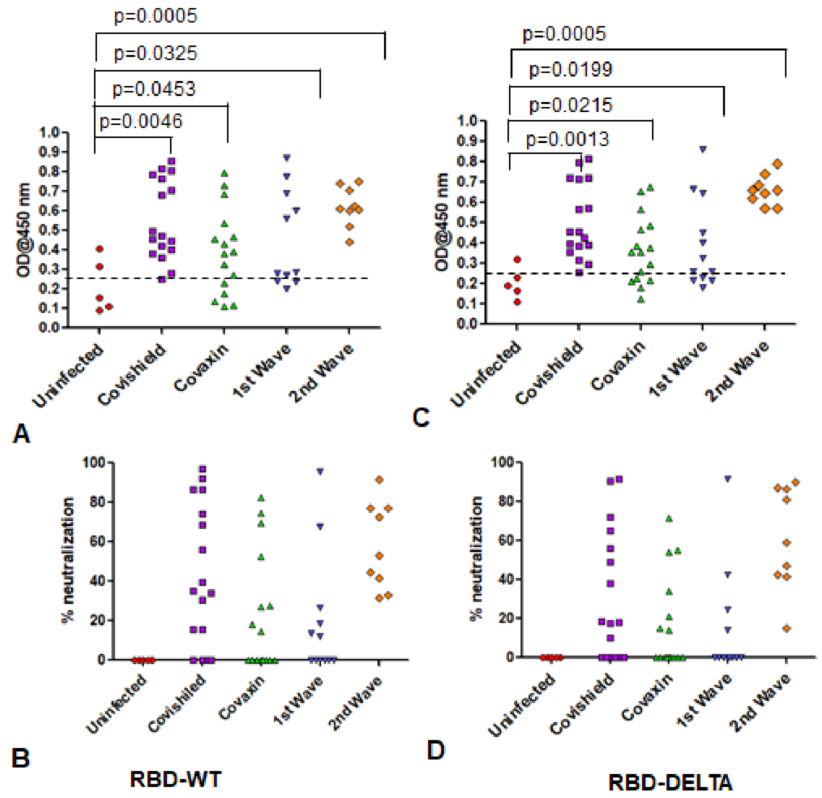


**Supplementary Figure 4:** Humoral immunity of samples of all five groups used for cell mediated immunity. **A** & **B:** Humoral immunity against RBD-WT. Seropositivity (A) and neutralization efficiency (B) against RBD-WT. **C** & **D:** Humoral immunity against RBD-DELTA. Seropositivity (C) and neutralization efficiency (D) against RBD-DELTA.

| Groups | Number of Subjects |
|---|---|
| Covishield | 16 |
| Covaxin | 16 |
| Infection 1st wave | 12 |
| Infection 2nd wave | 9 |
| Uninfected | 5 |

**Supplementary Table 1:** Summary of test subjects for assessment of cell mediated immunity
